# Modified Vaccinia Virus Ankara Preferentially Targets Antigen Presenting Cells *In Vitro*, *Ex Vivo* and *In Vivo*

**DOI:** 10.1038/s41598-017-08719-y

**Published:** 2017-08-17

**Authors:** Arwen F. Altenburg, Carolien E. van de Sandt, Bobby W. S. Li, Ronan J. MacLoughlin, Ron A. M. Fouchier, Geert van Amerongen, Asisa Volz, Rudi W. Hendriks, Rik L. de Swart, Gerd Sutter, Guus F. Rimmelzwaan, Rory D. de Vries

**Affiliations:** 1 Department of Viroscience, Postgraduate School of Molecular Medicine, Erasmus MC, Rotterdam, The Netherlands; 2 Department of Pulmonary Medicine, Erasmus MC, Rotterdam, The Netherlands; 3 Aerogen Ltd, IDA Business Park, Dangan, Galway Ireland; 4 ViroClinics Biosciences BV, Rotterdam, The Netherlands; 5 Institute for Infectious Diseases and Zoonoses, LMU University of Munich, Munich, Germany; 6 German Centre for Infection Research (DZIF) Inhoffenstraße 7, 38124 Braunschweig, Germany

## Abstract

Modified Vaccinia virus Ankara (MVA) is a promising vaccine vector with an excellent safety profile. However, despite extensive pre-clinical and clinical testing, surprisingly little is known about the cellular tropism of MVA, especially in relevant animal species. Here, we performed *in vitro*, *ex vivo* and *in vivo* experiments with recombinant MVA expressing green fluorescent protein (rMVA-GFP). In both human peripheral blood mononuclear cells and mouse lung explants, rMVA-GFP predominantly infected antigen presenting cells. Subsequent *in vivo* experiments performed in mice, ferrets and non-human primates indicated that preferential targeting of dendritic cells and alveolar macrophages was observed after respiratory administration, although subtle differences were observed between the respective animal species. Following intramuscular injection, rMVA-GFP was detected in interdigitating cells between myocytes, but also in myocytes themselves. These data are important in advancing our understanding of the basis for the immunogenicity of MVA-based vaccines and aid rational vaccine design and delivery strategies.

## Introduction

Modified Vaccinia virus Ankara (MVA) is an attenuated poxvirus that is frequently used as viral vector. MVA is derived from the chorioallantois vaccinia virus strain Ankara by serial passaging in chicken embryo fibroblasts (CEF) over 500 times. This resulted in major deletions in the viral genome and rendered MVA replication-deficient in mammalian cells^1^. MVA was used in smallpox vaccination regimens and has been tested in numerous clinical trials, resulting in the immunization of >100.000 study subjects without serious adverse events^2, 3^. Moreover, MVA-based vaccines also proved safe in immunocompromised non-human primates^4^. Given this impressive safety record, combined with the capacity to encode genes of interest of up to 10 kb in size, MVA holds promise as a vaccine vector. Vaccination with recombinant (r)MVA leads to efficient induction of both humoral and cellular immune responses targeting proteins encoded by the inserted transgene (reviewed in refs 5 and 6). Because of these favourable properties, there has been considerable interest in developing rMVA-based vaccines against various infectious diseases and cancer, reflected by the steady increase in the number of clinical trials that have been performed with rMVA in recent years^7^.

Despite frequent testing in clinical trials, the cellular tropism of MVA, particularly in relevant animal models, has been studied only to a limited extent. Even though the poxvirus lifecycle is complicated, in general poxviruses enter target cells via direct fusion with the cell membrane or endocytosis^8^, but the cellular receptor enabling either process has not been identified. Because MVA promiscuously infects almost any cell type, a putative cellular receptor is expected to be a ubiquitously expressed protein shared by different cell types^9^. Extensive research has been performed with vaccinia virus (VACV), the parental pathogenic and replication-competent poxvirus closely related to MVA, which implicated an important role for cell surface proteoglycans in VACV attachment^10, 11^. Identical or similar proteins could be involved in attachment and entry of MVA into target cells.

Recombinant viruses expressing fluorescent reporter proteins that can be sensitively traced *in vitro* and *in vivo* have been instrumental in improving our understanding of the tropism of different viruses^12–15^. Previous *in vitro* studies with human peripheral blood mononuclear cells (PBMC), performed to determine the cellular tropism of VACV, showed that recombinant VACV expressing green fluorescent protein (GFP) preferentially infected professional antigen-presenting cells (APC)^9, 16, 17^. In accordance with these results, similar *in vitro* infection studies with rMVA expressing GFP (rMVA-GFP) also demonstrated that APC were preferentially infected, directly followed by apoptosis of these target cells^18–20^. Furthermore, to determine the tissue tropism of MVA *in vivo*, mice were previously inoculated via the intranasal (IN), intraperitoneal (IP) or subcutaneous (SC) route with rMVA expressing luciferase^21, 22^. In these studies, it was shown that following IN instillation rMVA-infected cells could be detected in nasal-associated lymphoid tissue (NALT), lungs and draining lymph nodes (LN) of the lungs. Following IP and SC injection, rMVA-encoded luciferase was detected in all lymphoid organs, lungs, liver and ovaries. A mouse study with rMVA-GFP demonstrated predominant infection of CD11c^+^ dendritic cells (DC) in the spleen at 9 hour post- administration (HPA), however, in this study rMVA-GFP was administered intravenously^19^, which is not a standard immunization route. These studies focused on tissue tropism in mice, but the nature and phenotype of cells targeted by MVA *in vivo* in this and other, more relevant, animal models after administration via routes commonly used for vaccination, remain largely unknown.

In order to extensively elucidate the tissue- and cell tropism of MVA, we performed *in vitro*, *ex vivo* and *in vivo* infection studies with rMVA-GFP. In addition, we compared the *in vivo* cell tropism of MVA after IM injection with tropism after direct delivery to the respiratory tract. We demonstrated predominant infection of CD11c^+^ MHC class II^+^ DC by rMVA-GFP *in vitro* in human PBMC and *ex vivo* in mouse lung explants. *In vivo*, rMVA-GFP was detected in both interdigitating cells in between myocytes and myocytes themselves after IM injection, whereas direct administration of rMVA-GFP to the respiratory tract led to preferential targeting of alveolar macrophages (AM) in mice and DC in non-human primates, respectively. Since GFP^+^ cells were subsequently detected in the LN draining the site of administration, we concluded that rMVA-GFP-infected cells migrated to secondary lymphoid tissues and that direct targeting of professional APC is involved in the shaping of the immune response.

## Results

### rMVA-GFP predominantly infects professional APC *in vitro* and *ex vivo*

**Figure 1:**
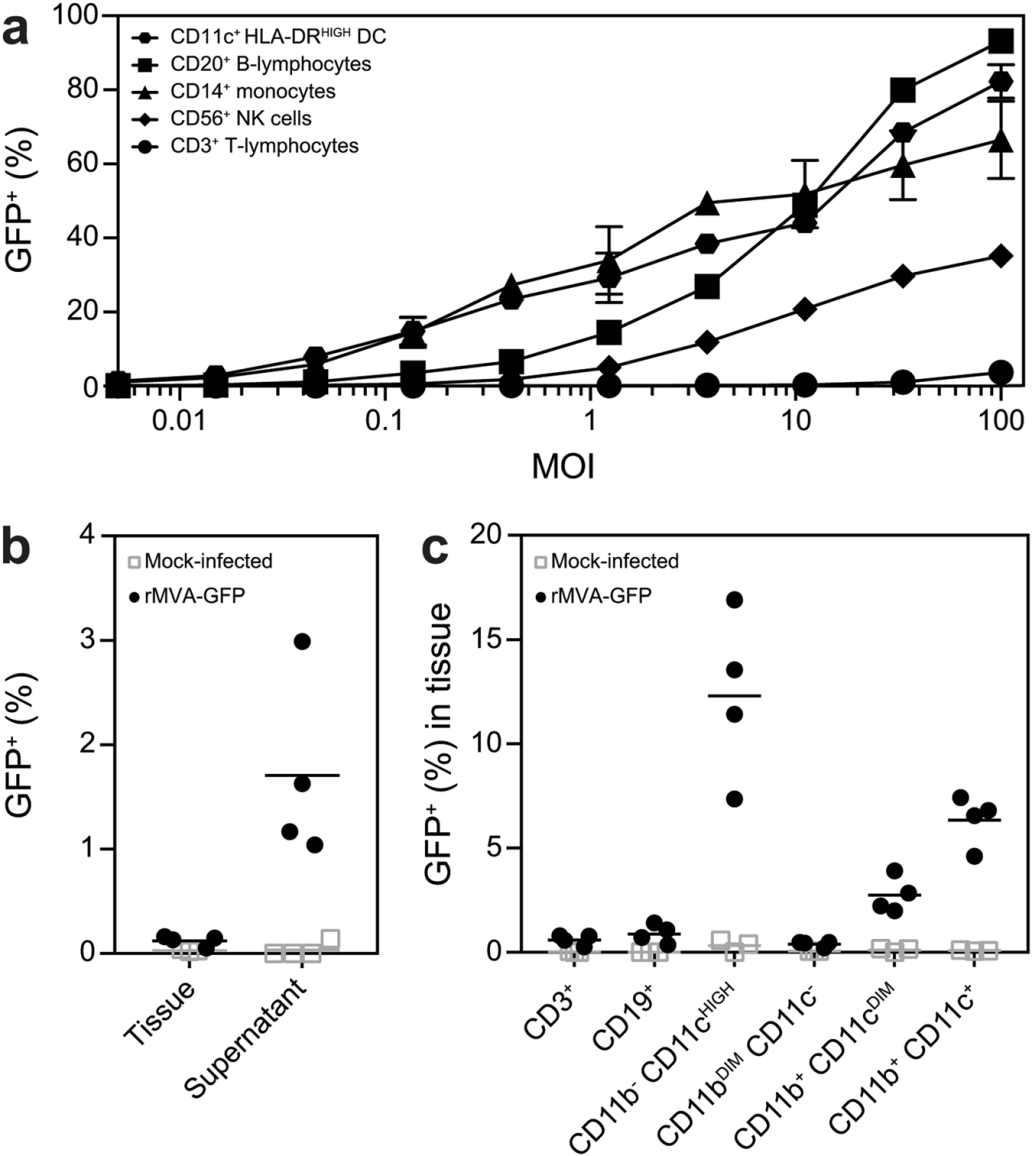
Populations infected by rMVA-GFP *in vitro* in human PBMC and *ex vivo* in mouse lung slices. (**a**) Human PBMC were inoculated with rMVA-GFP at various MOI. Percentage of GFP^+^ live cells within DC, B-lymphocyte, monocyte, NK cell and T-lymphocyte populations were determined by flow cytometry at 24 h post-infection. Mean of duplicates and standard deviation are indicated. (**b**) Lung slices were inoculated with rMVA-GFP and analysed by flow cytometry after 24 h. GFP^+^ cells in single cell suspensions of lung tissue and culture supernatant were detected. (**c**) GFP^+^ cells in single cell suspensions of lung slices were phenotyped by flow cytometry. Populations were defined as CD3^+^, CD19^+^, CD11b^−^ CD11c^HIGH^, CD11b^DIM^ CD11c^−^, CD11b^+^ CD11c^DIM^ or CD11b^+^ CD11c^+^. Mean percentage of infection per population of four lung slice cultures is indicated.

To determine the cellular tropism of MVA *in vitro*, human PBMC were inoculated with rMVA-GFP at incrementing multiplicity of infection (MOI), ranging from 0.01 to 100. The percentage GFP^+^ cells within different cell populations was determined by flow cytometry 24 h post-inoculation. CD11c^+^ HLA-DR^HIGH^ DC and CD14^+^ monocytes were readily infected by rMVA-GFP, even at low MOI. CD20^+^ HLA-DR^+^ B-lymphocytes were also easily infected by rMVA-GFP, followed by CD56^+^ NK cells. In contrast, CD3^+^ T-lymphocytes were refractory to infection with rMVA-GFP (Fig. 1a).

To determine MVA tropism in the respiratory tract, mouse lung slices were prepared and inoculated *ex vivo* with rMVA-GFP. At 24 h post-inoculation, both the emigrant cells in the lung slice culture supernatant and single cells suspensions of the lung tissue were analysed for the presence of GFP^+^ cells using flow cytometry. Interestingly, GFP^+^ cells were mainly present in the supernatant of inoculated lung slice cultures, however, could also be detected in the single cell suspensions (Fig. 1b). Phenotyping of GFP^+^ tissue-resident lung cells showed predominant rMVA-GFP infection of CD11b^−^ CD11c^high^ myeloid cells, commonly identified as AM or CD103^+^ DC^23^. However, CD11c^+^ and CD11c^dim^ myeloid cells double positive for CD11b^+^ were also infected (Fig. 1c). CD19^+^ B-lymphocytes were infected to a limited extent, whereas CD11b^dim^ CD11c^−^ single positive cells and CD3^+^ T-lymphocytes were found refractory to infection (Fig. 1c).

### rMVA-GFP^+^ tissues and cells after IM or respiratory administration *in vivo*

**Figure 2:**
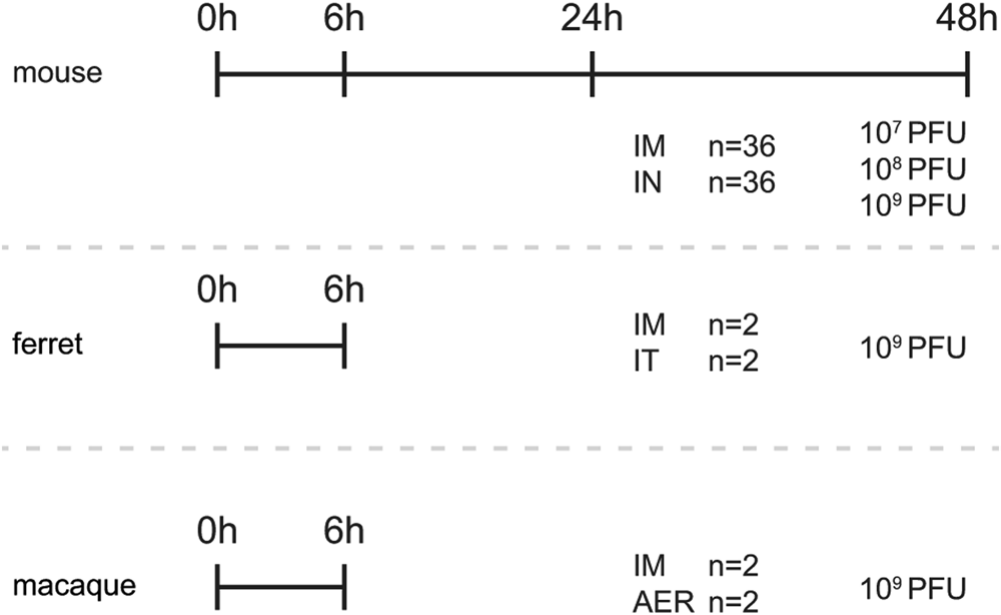
Experimental setup of rMVA-GFP *in vivo* studies. Three different doses (10^7^–10^9^ PFU) of rMVA-GFP were administered to mice via IM injection or IN instillation. At 6, 24 or 48 HPA, mice were euthanized. Ferrets and macaques received 10^9^ rMVA-GFP via IM injection or direct respiratory tract administration (IT inoculation for ferrets, AER inhalation for macaques) and were euthanized 6 HPA.

In order to determine the tissue and cell tropism of MVA *in vivo*, rMVA-GFP was administered IM and to the respiratory tract of three different animal species (mice, ferrets and non-human primates, Fig. 2). Respiratory tract administration was performed by IN instillation, intra-tracheal (IT) inoculation or aerosol (AER) inhalation, depending on the animal model. Importantly, all routes in the respective animal models allowed for rMVA-GFP deposition in the lower respiratory tract. First, 10^7^, 10^8^ or 10^9^ plaque forming units (PFU) rMVA-GFP was administered to C57BL/6 mice via the IM or IN route. 6, 24 or 48 HPA mice were sacrificed to determine the optimal dose and time-point for follow-up experiments in ferrets and non-human primates.

### rMVA-GFP targets myocytes and interdigitating cells after IM injection of mice

**Figure 3:**
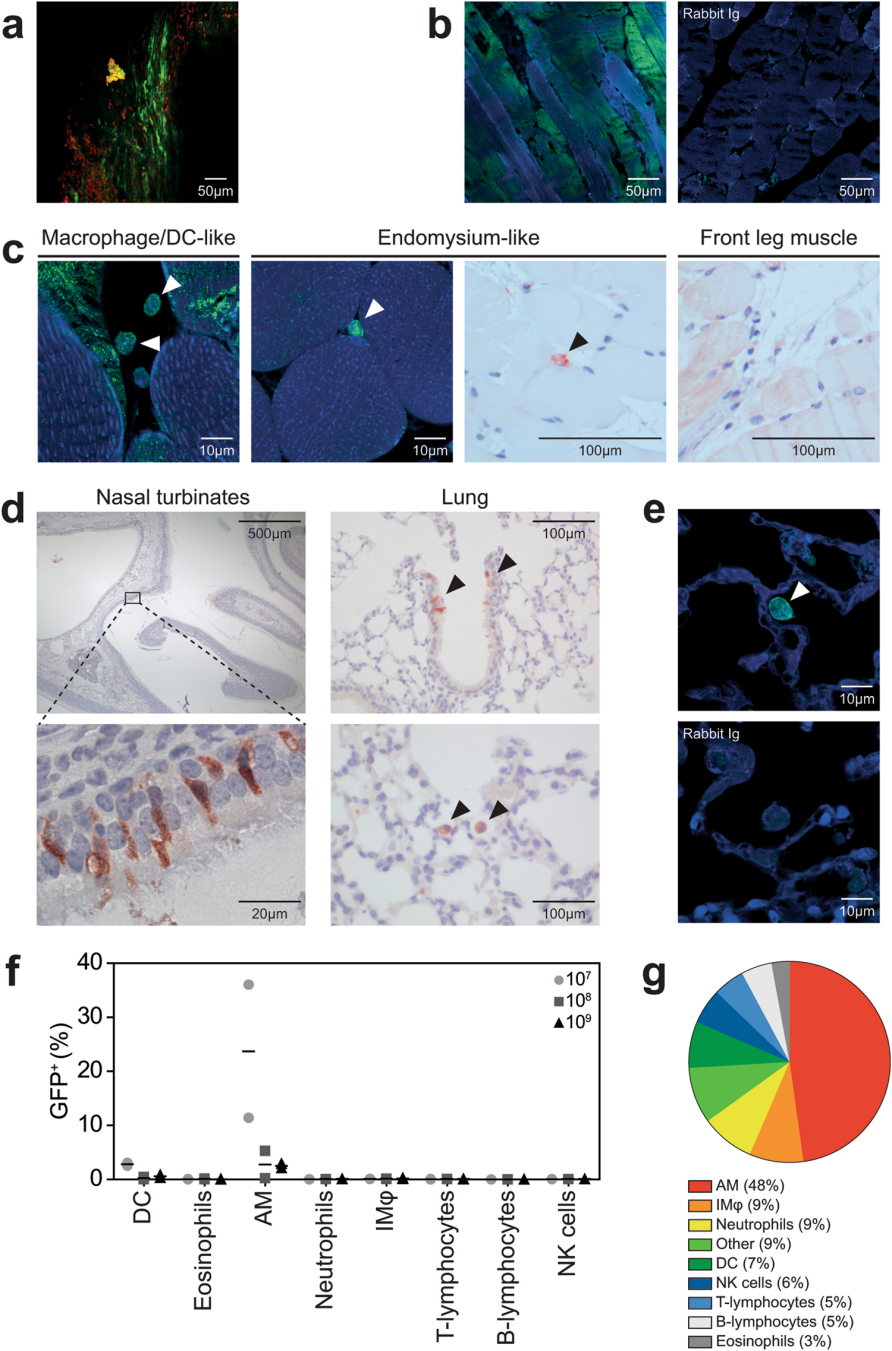
GFP^+^ cells in muscle or respiratory tract after IM injection or IN instillation of rMVA-GFP to mice. (**a**) Maximum intensity projection of a Z-stack of the hind leg muscle 24 h after IM injection of rMVA-GFP. Green = GFP. Red = nucleus (stained by TO-PRO3). (**b**) Detection of GFP^+^ myocytes in hind leg muscle tissue after IM rMVA-GFP injection by staining with rabbit anti-GFP or rabbit Ig isotype (green) in combination with DAPI (blue). (**c**) Morphological characterization of GFP^+^ cells in hind leg muscle tissue after IM rMVA-GFP injection by CLSM (left two panels) or IHC (right two panels) showing hind leg muscle (24 HPA) or negative control front leg muscle (6 HPA). GFP^+^ interdigitating cells are indicated by arrows. CLSM: GFP = green, nucleus = blue (DAPI). IHC: GFP = red and counterstaining with haematoxylin. (**d**) Detection of GFP^+^ cells in the nasal turbinates and lungs 6 or 24 h after IN instillation of rMVA-GFP. GFP^+^ cells are shown in red (indicated by arrows). Tissues were counterstained with haematoxylin. (**e**) Morphological characterization of GFP^+^ cells in lungs after IN instillation of rMVA-GFP by staining with rabbit anti-GFP or rabbit Ig isotype (green) in combination with DAPI (blue). GFP^+^ cell with macrophage-/DC-like morphology is indicated by arrow. (**f**) Percentage of GFP^+^ cells within different cell populations 6 h after IN instillation of 10^7^, 10^8^ or 10^9^ PFU rMVA-GFP determined by flow cytometry. Results are shown as mean of two mice. (**g**) Relative contribution of the different cellular subsets to the GFP^+^ population by reversed gating. The average percentages of all mice that received rMVA-GFP by IN instillation and were euthanized 6 HPA are shown. IMφ = interstitial macrophages﻿. Contrast of some CLSM images have been linearly enhanced using Adobe Photoshop CC. Scale bars are indicated in each figure.

After IM injection of rMVA-GFP in mice, GFP^+^ cells were consistently detected in the hind leg muscle in which rMVA-GFP was injected. Direct confocal laser scanning microscopy (CLSM) of muscle slices resulted in detection of GFP fluorescence in the muscle fibers (Fig. 3a and Supplementary Figure 1). Phenotypic characterization by dual staining and subsequent CLSM and immunohistochemistry (IHC) proved difficult for mouse tissues, however, myocytes were clearly infected (Fig. 3b). GFP was never found in the isotype control staining of hind leg muscle (Fig. 3b). Furthermore, GFP^+^ interdigitating cells were detected in the muscles and were identified as either having macrophage-/DC-like morphology or as part of the endomysium, the connective tissue that separates muscle fibers (Fig. 3c). Using IHC, GFP^+^ cells were never found in the front leg muscle, which was used as a negative control (Fig. 3c).

### rMVA-GFP targets predominantly AM in mice after IN instillation

At 6 or 24 h after IN instillation of rMVA-GFP in mice, GFP^+^ cells were detected in both the nasal turbinates and the lungs. Mice were inoculated with a relatively large volume, directly exposing both the nasal cavity and the lungs. In the nasal turbinates, GFP^+^ cells were exclusively observed in the epithelial layer and had the morphology of epithelial cells. In the lungs, GFP^+^ cells were also detected in the epithelium of bronchioles, however, single GFP^+^ cells with an AM-like morphology were also observed in the lumen of the alveoli by IHC and CLSM (Fig. 3d,e). These data show that rMVA-GFP^+^ cells can consistently be detected at the anatomical site of administration and the infected cells morphologically resemble epithelial cells or AM.

Lungs of mice inoculated via the IN route were obtained during necropsy and single cell suspensions were prepared to determine the phenotype of GFP^+^ cells by multicolour flow cytometry (Supplementary Figure 2). GFP^+^ cells in the lungs were predominantly AM (defined as CD3^−^, CD19^−^, NK1.1^−^, Siglec-F^+^, CD11c^+^, F4/80^+^ and CD11b^+^), however, GFP^+^ DC (defined as CD3^−^. CD19^−^, NK1.1^−^, MHC class II^+^ and CD11c^+^) were also detected albeit to a lesser extent (Fig. 3e). Similar results were observed 24 HPA, whereas no GFP^+^ cells could be detected in the lungs 48 HPA (data not shown). Notably, when all GFP^+^ cells were selected at 6 HPA and subsequently characterized on basis of expression of various surface markers by reverse gating, it was found that AM constituted the largest proportion of GFP^+^ cells (48%, Fig. 3f).

### rMVA-GFP^+^ cells migrate to the draining LN and spread systemically in mice

To determine whether rMVA-GFP-infected cells migrated to the draining LN, the inguinal LN (ING-LN, draining the hind leg muscle) or mediastinal LN (MED-LN, draining the chest) was examined for presence of GFP^+^ cells after IM injection or IN instillation, respectively. As internal negative control the axillary (AX-LN) draining the front leg muscle (after IM injection) or ING-LN (after IN instillation) were used. To determine whether rMVA-GFP could be detected systemically, blood and spleen samples were analysed for the presence of GFP^+^ cells.

**Figure 4:**
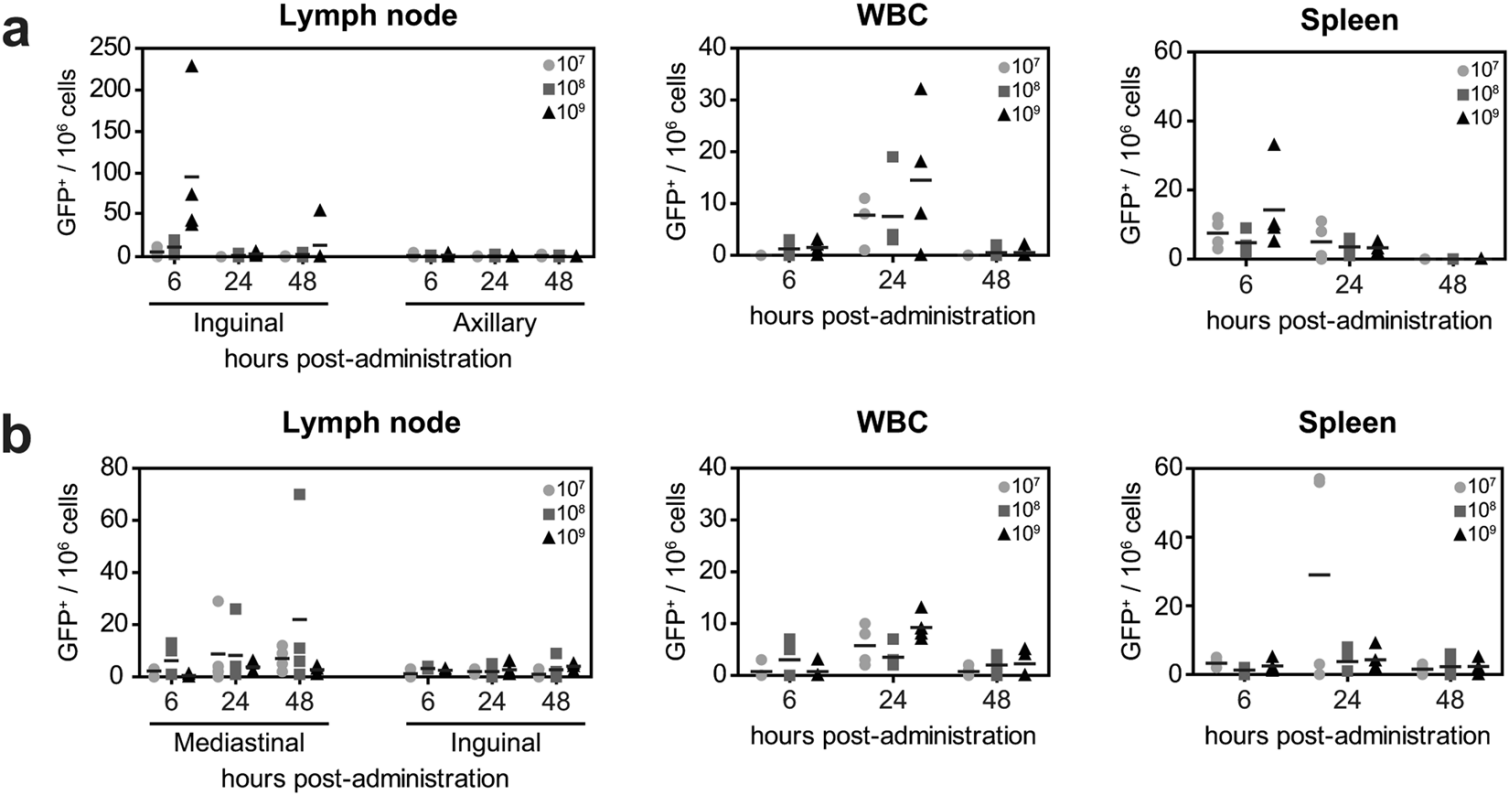
Dissemination of GFP^+^ cells after IM injection or IN instillation of rMVA-GFP to mice. At 6, 24 or 48 HPA of 10^7^, 10^8^ or 10^9^ PFU rMVA-GFP administration by IM injection (**a**) or IN instillation (**b**), GFP^+^ cells were detected in LN, WBC and spleens using flow cytometry. Number of GFP^+^ cells per 10^6^ total cells is shown, mean of four mice is indicated.

After IM injection, GFP^+^ cells were detected in single cell suspensions of the draining ING-LN as early as 6 HPA, mainly in mice that received the highest dose (10^9^ PFU) rMVA-GFP. GFP^+^ cells were hardly detected at later time-points and were never observed in the control AX-LN (Fig. 4a, left panel). Notably, GFP^+^ cells were also detected systemically; in white blood cells (WBC) and single cell suspensions of the spleen at 6 and 24 HPA (Fig. 4a). After IN instillation of rMVA-GFP, low numbers of GFP^+^ cells were detected in the MED-LN at all time-points, but not in the control ING-LN (Fig. 4b, left panel). Similar to IM rMVA-GFP injection, GFP^+^ cells were detected systemically in WBC and spleen after IN instillation (Fig. 4b). Collectively, these data illustrate that both after IM injection and IN instillation, GFP^+^ cells can be detected in the LN draining the site of inoculation and systemically.

### rMVA-GFP infected cells in ferrets are of DC- or macrophage-like morphology

**Figure 5:**
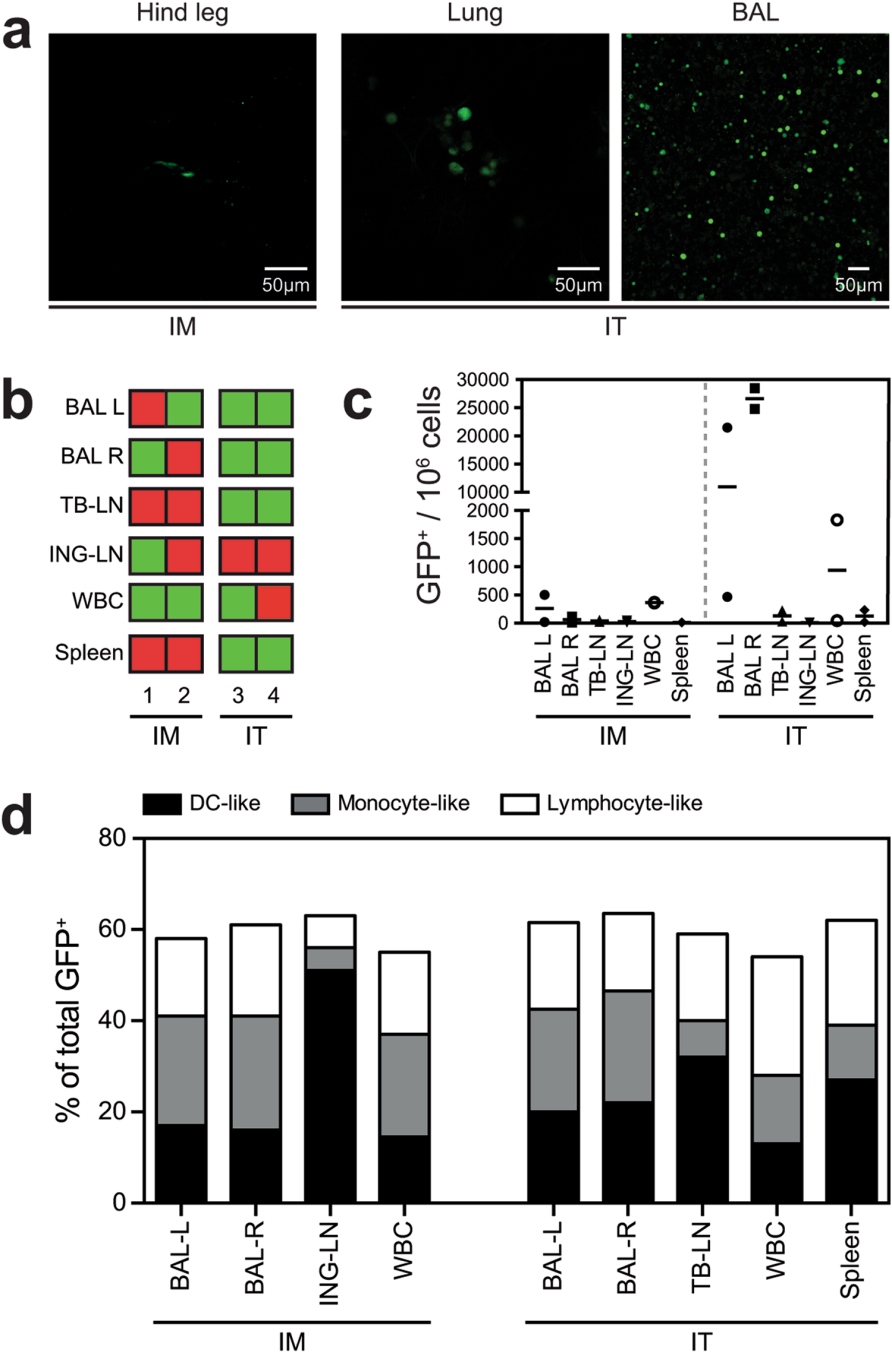
Detection of GFP^+^ cells in ferrets after IM injection and IT inoculation of rMVA-GFP. (**a**) Direct detection of GFP^+^ cells using CLSM after IM injection (left panel) or IT inoculation (middle and right panel) of 10^9^ PFU rMVA-GFP, 6 HPA. Middle panel shows direct CLSM of a lung slice, whereas the right panel shows cells recovered from the lungs by BAL. Contrast of certain images was linearly enhanced using Adobe Photoshop CC. (**b**) Overview of detection of GFP^+^ cells in BAL (left [L] or right [R] lung lobes), TB-LN, ING-LN, WBC or spleen in each individual ferret (n = 2 per group) after IM injection or IT inoculation of rMVA-GFP. Green = GFP^+^. Red = GFP-negative. (**c**) Frequency of GFP^+^ cells in the respective tissues after IM injection or IT inoculation of rMVA-GFP. Number of GFP^+^ cells per 10^6^ total cells is shown, mean of two ferrets is indicated. (**d**) GFP^+^ cells were phenotypically characterized as DC-like, monocyte-like or lymphocyte-like cells based on scatter properties of the different cell types.

After determination of the optimal dose and kinetics of GFP expression in mice, ferrets received a high dose (10^9^ PFU) of rMVA-GFP via either IM injection or IT inoculation and were sacrificed at 6 HPA. GFP^+^ cells were detected in hind leg muscle slices after IM injection and lung slices and broncho-alveolar lavage (BAL) cells after IT inoculation, respectively (Fig. 5a). Although direct CLSM could not be used to determine the phenotype of GFP^+^ cells, it demonstrated that rMVA-GFP infected cells were present at the site of administration at 6 HPA. BAL cells, WBC and single cells suspensions of lymphoid tissues obtained from ferrets were directly analysed for GFP expression by flow cytometry. By performing positive/negative scorings based on flow cytometry data (Fig. 5b), BAL cells were consistently positive after IT inoculation and GFP^+^ cells disseminated from the site of administration and could be detected in the ING-LN draining the hind leg muscle and trachea-bronchial (TB-)LN draining the lungs after IM injection or IT inoculation, respectively (Fig. 5b,c). Systemic rMVA-GFP^+^ cells were detected in both the spleen and WBC of multiple animals, however, the detection was not consistent. Low frequencies of GFP^+^ cells were found in BAL after IM injection, potentially due to the circulation of GFP^+^ cells in these animals (reflected by GFP^+^ cells in PBMC). Of note, some tissues that scored positive in flow cytometry (Fig. 5b) appear negative in Fig. 5c, due to the low frequency of GFP^+^ events. Supplementary Figure 3 illustrates when tissues were scored positive using flow cytometry.

Phenotyping of GFP^+^ cells in ferrets was experimentally challenging due to lack of the required ferret-specific antibodies directed against discriminative surface antigens. Therefore, we reversely gated the GFP^+^ events observed in flow cytometry and discriminated between three different populations based on known scatter properties of different cell types: lymphocyte-like, monocyte-like and DC-like cells (Supplementary Figure 3). Notably, GFP^+^ cells were equally distributed over the different populations for BAL, WBC and splenocytes, but there was a clear preference for DC-like cells in the draining LN of the hind leg muscle or lung after IM injection or IT inoculation, respectively. This suggests migration of rMVA-GFP-infected DC from the site of administration to the draining LN (Fig. 5d).

### Validation of rMVA-GFP aerosol for administration to non-human primates

During aerosolization, an aerosol plume consisting of a droplet size distribution was generated. From within this plume, smaller droplets have a tendency towards deposition in the lower airways, whereas larger droplets most likely deposit in the nasal cavity and conducting airways. In order to fully characterize the aerosol and predict where in the respiratory tract rMVA-GFP may deposit, cascade impaction was performed during which the aerosol is fractioned into discrete droplet size ranges between 0.98 and 14.1 μm. Each fraction was titrated in order to determine in what size aerosol droplets viable rMVA-GFP is present (Supplementary Figure 4). The Fine Particle Fraction (FPF) - the percentage of the rMVA particles contained in droplets under 5 μm - was calculated as 96.75% with a mass median aerodynamic diameter (MMAD) of 1.70 μm, predicting targeting of the lower airways of non-human primates. However, through the use of a facemask interface it was ensured that the nasal cavity/upper airways were exposed to aerosol as well. Collectively, these results confirm that rMVA-GFP can be delivered to the upper and lower respiratory tract of non-human primates via aerosol inhalation.

### rMVA-GFP predominantly infects myocytes, DC and AM in non-human primates

**Figure 6:**
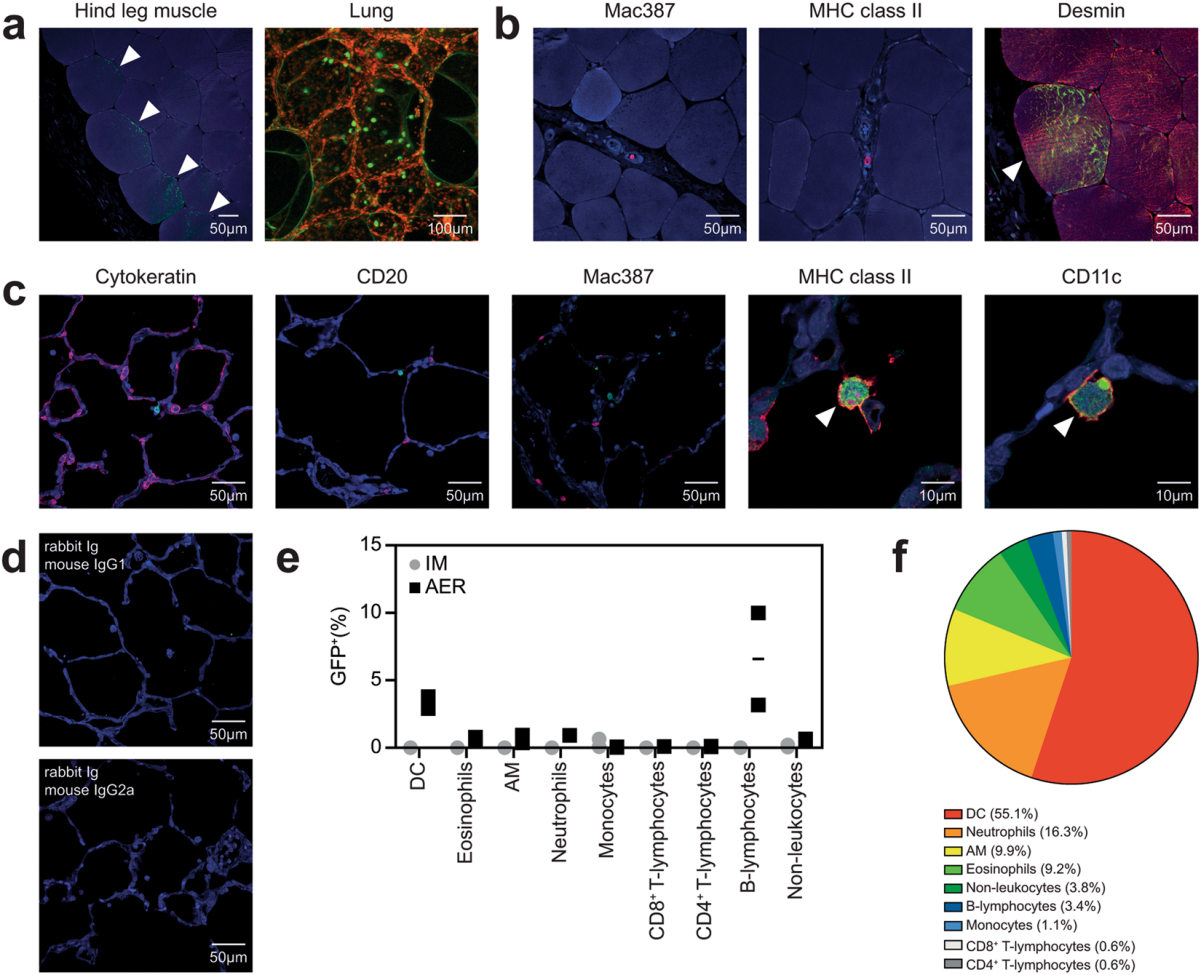
Identification of rMVA-GFP^+^ cells in macaque muscle or lung after IM injection or AER inhalation, respectively. (**a**) Detection of GFP^+^ cells at site of rMVA-GFP administration. Left panel: hind leg muscle after IM injection stained with rabbit anti-GFP (green, indicated by arrows) and DAPI (blue). Right panel: direct detection of GFP fluorescence in lung slices after AER inhalation counterstained with TO-PRO3 (red). Maximum intensity projection of a Z-stack is shown. (**b**) Dual immunofluorescent staining of muscle slices after IM injection of rMVA-GFP with rabbit anti-GFP (green) in combination with DAPI (blue) and mouse anti-Mac387, anti-MHC class II or anti-desmin (red). Co-localization of GFP and desmin is indicated by an arrow. (**c**) Dual immunofluorescent staining of lung slices after AER inhalation of rMVA-GFP with rabbit anti-GFP (green) in combination with DAPI (blue) and mouse anti-cytokeratin, anti-CD20, anti-Mac387, anti-MHC class II or anti-CD11c (red). Co-localization of GFP and MHC class II or CD11c is indicated by arrows. (**d**) Lung slices stained with isotype control antibodies (rabbit Ig as control for anti-GFP, mouse IgG1 and 2a as control for cytokeratin, CD20, Mac387, MHC class II, CD11c, CD3 and desmin). (**e**) Percentage of GFP^+^ BAL cells within different cell populations after IM injection or AER inhalation of rMVA-GFP determined by flow cytometry. Mean of two macaques is indicated. (**f**) Relative contribution of the different subsets to the GFP^+^ population determined by reversed gating. The averages of the two animals that received rMVA-GFP by AER are shown. Contrast of some CLSM images has been enhanced linearly using Adobe Photoshop CC. Scale bars are indicated in images.

After completion of ferret experiments, cynomolgus macaques were inoculated with 10^9^ PFU rMVA-GFP by IM injection or aerosol (AER) inhalation^24–26^. Slices from either the hind leg muscle or lungs obtained from non-human primates at necropsy 6 HPA were analysed by direct CLSM, which revealed presence of GFP^+^ cells after IM injection or AER inhalation, respectively (Fig. 6a). 3D reconstruction of a Z-stack obtained by CLSM of a non-human primate lung slice revealed that GFP^+^ cells were predominantly detected in the lumen of the alveoli, and to a lesser extent directly in or connected to the epithelium lining the alveoli (Fig. 6a right panel, Supplementary Figure 5).

Direct imaging of muscle and lung slices by CLSM did not allow identification of the phenotype of GFP^+^ cells, but guided collection of GFP^+^ tissues that could be used for subsequent dual immunofluorescent staining. Mac387^+^ interstitial macrophages (IMφ) and MHC class II^+^ antigen presenting cells were only detected infrequently in the muscles and co-localization with GFP could not be observed in non-human primates that received rMVA-GFP by IM injection. However, GFP mainly co-localized with desmin, a specific marker for myocytes (Fig. 6b). Dual immunofluorescent staining of GFP^+^ lung slices of non-human primates that were inoculated by AER inhalation revealed co-localization with MHC class II^+^ and CD11c^+^ APC (Fig. 6c). Morphologically, these cells resembled DC and AM. Co-localization of GFP with specific markers for T-lymphocytes (CD3, not shown), epithelial cells (cytokeratin), B-lymphocytes (CD20) or IMφ (Mac387) was not observed after AER inhalation (Fig. 6c). Isotype control stainings of lung slices were negative (Fig. 6d).

Subsequently, BAL samples from non-human primates inoculated by AER were analysed by multicolour flow cytometry. DC (CD45^+^ HLA-DR^+^ Siglec-8^−^ CD16^−^ CD11b^+^ CD11c^+^), eosinophils (CD45^+^ HLA-DR^+^ Siglec-8^+^ CD16^+^), AM (CD45^+^ HLA-DR^+^ Siglec-8^−^ CD16^−^ CD11b^+^ CD11c^−^), neutrophils (CD45^+^ HLA-DR^+^ Siglec-8^−^ CD16^+^), B-lymphocytes (CD45^+^ CD3^−^ HLA-DR^+^), CD4^+^/CD8^+^ T-lymphocytes (CD45^+^ CD3^+^) and non-leukocytes (CD45^−^) were analysed for GFP expression (Supplementary Figure 6). Respiratory samples collected after IM injection were used as negative control and indeed GFP^+^ cells were not detected (Fig. 6e). The relative percentage of GFP^+^ cells within subsets was highest in B-lymphocytes and DCs (Fig. 6e). In addition, GFP^+^ eosinophils, AM, neutrophils and non-leukocytes, probably representing epithelial cells, were detected (Fig. 6e). Reverse gating of GFP^+^ cells showed that DCs constituted 55.1% of the GFP^+^ cells and were the largest GFP^+^ cell population. Interestingly, although B-lymphocytes showed the highest rMVA-GFP infection rate, the number of B-lymphocytes recovered from non-human primate lungs was low, which explains the low contribution of B-lymphocytes to the total GFP^+^ cell population (Fig. 6e,f).

### rMVA-GFP^+^ cells migrate to the draining LN in non-human primates

**Figure 7:**
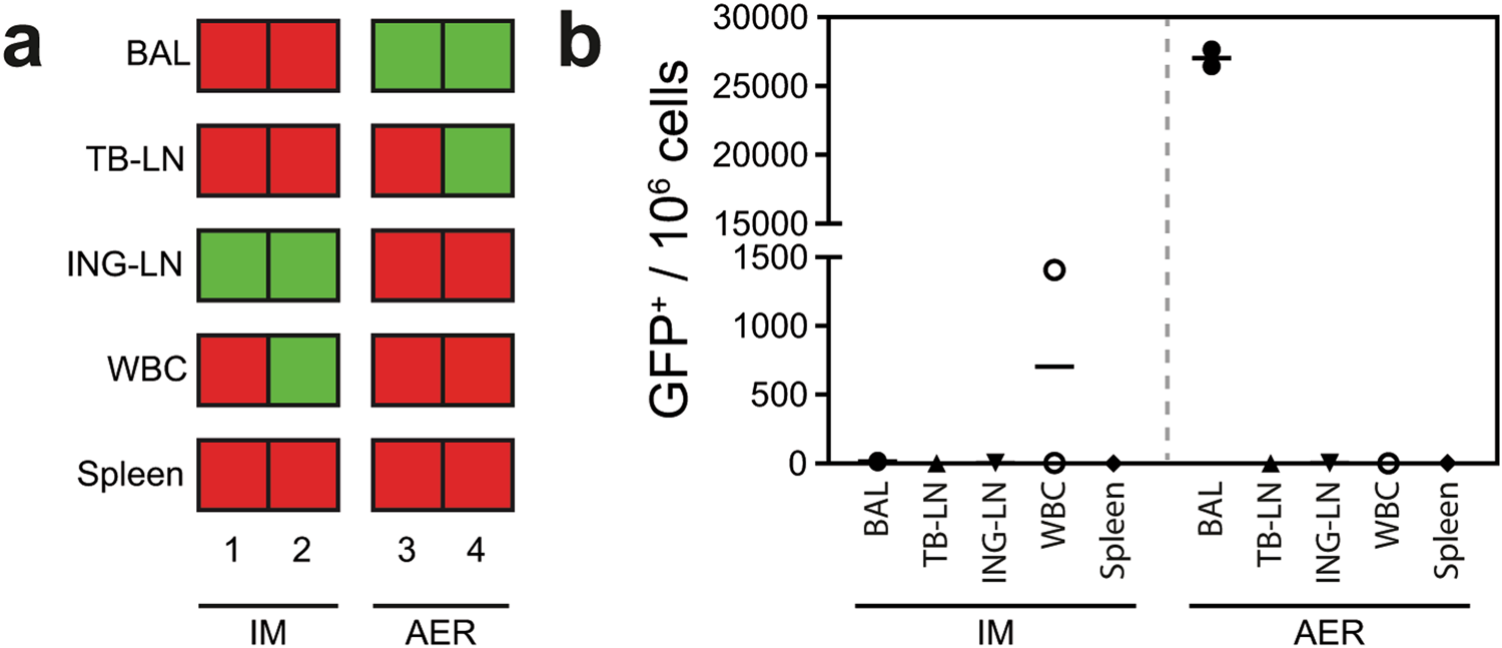
Dissemination of rMVA-GFP^+^ cells in macaques. (**a**) Overview of GFP^+^ cells detected in BAL, TB-LN, ING-LN, WBC or spleen in each individual macaque (n = 2 per group) after IM injection or AER inhalation of rMVA-GFP. Green = GFP^+^. Red = GFP-negative. (**b**) Frequency of GFP^+^ cells in the respective tissues after IM injection or AER inhalation of rMVA-GFP. Number of GFP^+^ cells per 10^6^ total cells is shown, mean of two macaques is indicated.

To determine whether rMVA-GFP^+^ cells in non-human primates migrated to draining LN and spread systemically, WBC and single cell suspensions of the LN and spleen were analysed by flow cytometry. By performing positive/negative scorings based on flow cytometry data of the different tissues, we found that the BAL of AER inoculated macaques was consistently positive, and showed that GFP^+^ cells had migrated to the draining ING-LN after IM injection (2/2 non-human primates, Fig. 7a). However, the TB-LN was not consistently GFP^+^ after AER inhalation (1/2 non-human primates, Fig. 7a). GFP^+^ cells were never detected in the spleen, and GFP^+^ WBC were only observed after IM injection in 1/2 animals (Fig. 7a). Thus, apart from migration of MVA-infected cells to the draining LN, systemic spread of GFP^+^ cells was detected to a limited extent (Fig. 7). Of note, some tissues that scored positive in flow cytometry (Fig. 7a) appear negative in Fig. 7b, due to the low frequency of GFP^+^ events. Supplementary Figure 6 illustrates when tissues were scored positive using flow cytometry.

## Discussion

In this study, we systematically assessed tissue and cell tropism of the vaccine vector MVA *in vitro*, *ex vivo* and *in vivo* using an rMVA expressing a fluorescent reporter protein. Even though different animal species and administration routes were used, we demonstrated consistent predominant infection of MHC class II^+^ APCs. In addition, in the different species local myocytes and local epithelial cells were infected after either IM injection or respiratory administration, respectively.

Vaccination is the cornerstone control measure to reduce morbidity and mortality caused by infectious diseases. However, vaccination against hypervariable and emerging pathogens can be challenging. For example, seasonal influenza vaccines need to be updated almost annually to antigenically match emerging drift variant viruses and to maintain vaccine efficacy. Furthermore, in case of an emerging pandemic strain of influenza virus, timely production of a pandemic influenza vaccine has proven to be challenging^27, 28^. The rapid construction of recombinant viral vaccine vectors, such as MVA, which allows easy insertion of any or multiple antigens of choice and rapid production of a large number of vaccine doses, is an attractive alternative to conventional vaccine production technology^29, 30^. In addition, MVA has been considered as vaccine delivery system for many other infectious diseases and cancers^31, 32^. Despite the increasing number of successful clinical trials performed with rMVA-based vaccines in recent years^7^, surprisingly little is known about their mode of action and the *in vivo* tissue and cell tropism of MVA, particularly in the ferret model, the golden standard for influenza virus pathogenesis studies, and animal models more closely related to humans such as non-human primates. This gap of knowledge could potentially hamper future registration of MVA-based vaccines for use in humans.

In this study, we describe the use of rMVA-GFP, allowing detection of single rMVA-GFP infected cells with high sensitivity, to elucidate the tropism of the viral vector MVA. *In vitro* tropism was investigated by inoculating human PBMC with rMVA-GFP, *ex vivo* tropism by inoculation of mouse lung explants and *in vivo* tropism by inoculating three different animal species via two different administration routes. In general, myeloid cells (mainly DC and macrophages) were preferentially infected by rMVA-GFP, independent of the experimental set up, animal species or route of administration. Direct targeting of APC in mice, ferrets and non-human primates confirms the suitability of MVA as vaccine vector, since they are involved in shaping the immune response. Of note, the relation between vector tropism and vaccine immunogenicity is not always straightforward, for example optimizing DC infection by an adenovirus vector did not result in increased immunogenicity in non-human primates^33^.

Upon *in vitro* inoculation of human PBMC with rMVA-GFP, DCs, monocytes and B-lymphocytes - all expressing MHC class II - were predominantly infected. NK cells were also targeted by rMVA-GFP (Fig. 1a) but T-lymphocytes were refractory to MVA infection, which was in accordance with previously published data obtained with both VACV and MVA^17–19^. In addition, the tropism of rMVA-GFP was assessed *ex vivo* in the respiratory tract in mouse lung slices. Due to limited use of surface markers, discrimination of distinct DC and monocyte populations was not possible. However, GFP^+^ cells were mainly identified in CD11c^high^ cells, most likely AM or CD103^+^ DC^23^ (Fig. 1c). Although these *in vitro* and *ex vivo* studies to elucidate the cellular tropism of MVA are of interest, they may not accurately reflect the *in vivo* situation and the cells infected with MVA at the site of administration.

Previous *in vivo* studies mainly focused on the tissue tropism of MVA^21, 22, 34^, whereas this study focused on the cellular tropism. *In vivo* administration of rMVA-GFP to mice, ferrets and non-human primates - via the IM or respiratory route - consistently resulted in the detection of GFP^+^ cells at the site of administration. In pre-clinical and clinical studies MVA is most commonly administered by IM injection, however, the local cell types that are initially infected by MVA have thus far not been identified. Here we showed that directly after IM injection, rMVA-GFP infected both myocytes and interdigitating cells, potentially macrophages, DC or part of the endomysium (Figs 3, 5 and 6). Unfortunately, we were unable to determine the exact phenotype the rMVA-GFP-infected interdigitating cells in non-human primates due to the low frequency of GFP^+^ cells in the muscle tissue.

Due to sensitive detection of GFP^+^ cells in single cell suspensions of lungs or BAL cells, identification of rMVA-GFP^+^ cells after respiratory administration of rMVA-GFP was feasible. IN instillation of mice with a relative large volume rMVA-GFP delivered the vector both to nasal turbinates and lungs. Consequently, MVA-infected epithelial cells were detected in the nasal turbinates and lungs, however, AM in the lungs were also frequently infected (Fig. 3). This is in concordance with results obtained in other studies, which showed that IN instillation of mice with rMVA-GFP resulted in the infection of CD11c^+^ MHC class II^−^ AM detected in BAL^35^. Targeting of myeloid cells was also observed after IT inoculation of ferrets with rMVA-GFP (Fig. 5), however, lack of ferret-specific antibodies required to distinguish between cell populations hampered the possibility to determine the exact phenotype of these rMVA-GFP-infected cells.

In contrast to mice, the predominant target cells for rMVA-GFP in the lungs of non-human primates were not characterized as CD11c^+^ MHC class II^−^ AM, but as CD11c^+^ MHC class II^+^ DC. This was shown by both flow cytometry of BAL cells and *in situ* by CLSM after dual immunofluorescence staining of lung tissue (Fig. 6). In addition to preferential targeting of DC, rMVA-GFP was also detected in AM, CD45^−^ epithelial cells, eosinophils and neutrophils by flow cytometry of BAL cells. The highest infection rate was observed in B-lymphocytes, but the numbers of B-lymphocytes present in BAL obtained from non-human primates were low (Fig. 6). DC largely contributed to the total number of rMVA-GFP^+^ cells, but neutrophils also formed a substantial component. Interestingly, it has been shown that after intradermal injection of MVA in mice, neutrophils become infected by MVA and subsequently migrate to the draining LN and bone marrow where they can prime phenotypically different CD8^+^ T-lymphocyte populations^36^.

In addition to detection at the site of administration, GFP^+^ cells were also detected in the respective draining LN after either IM injection or administration into the airways as early as 6 HPA. As the only exception, IN instillation of mice led to detection of GFP^+^ cells in the draining LN at later time-points (24–48 HPA, Fig. 4), possibly because the MED-LN were tested in this species instead of the TB-LN, which could not be harvested during necropsy of mice. Taken together, these data suggest that after administration, MVA locally infects APC that subsequently migrate to the draining LN. In addition, GFP^+^ cells were detected systemically in both blood and spleen in mice, particularly at 24 HPA (Fig. 4). Ferrets and non-human primates were euthanized 6 HPA, which could explain why systemic spread of MVA-GFP^+^ cells was observed only to a limited extent (Figs 5 and 7).

The standard route for administration of MVA-based vaccines is intramuscular (IM) injection. However, for the induction of protective mucosal immunity against respiratory pathogens, delivery of MVA to the respiratory tract has been considered (reviewed in ref. 37)^24, 38, 39^. In this study, rMVA-GFP was administered to the respiratory tract of non-human primates as an aerosol, as best possible proxy for future studies with MVA-based respiratory vaccines. Importantly, the aerosol droplet size and nebulizer delivery system chosen in this study allowed exposure of the entire upper and lower respiratory tract to infectious virus, allowing accurate definition of target cells (Supplementary Figure 4). Furthermore, aerosol delivery is under extensive investigation as an alternative for vaccine delivery by injection, as it abolishes the necessity for hypodermic needles. This facilitates safety of large-scale vaccination campaigns in developing countries with high prevalence of blood-borne infections, as there is no need for a complicated infrastructure to dispose of potentially contaminated needles. Finally, direct administration of vaccines to the respiratory tract could lead to efficient induction of local immune responses leading to increased protection from respiratory pathogens. This has been evaluated extensively with a live-attenuated influenza vaccine administered IN to children (FluMist®), showing that nasal spray administration led to efficient induction of local mucosal IgA antibody responses^38, 40, 41^. Similar advantages potentially exist when rMVA is used as live vaccine against respiratory pathogens and could lead to the induction of local humoral and cellular immune responses (tissue-resident T-lymphocytes)^39, 42^.

In summary, we are the first to comprehensively examine the tropism of MVA *in vitro*, *ex vivo* and *in vivo* in different species. Preferential targeting of APCs by rMVA-GFP was demonstrated *in vivo* in each animal model, regardless of the route of administration. Furthermore, delivery of rMVA-GFP to the respiratory tract of non-human primates as an aerosol also resulted in infection of local epithelial cells and IM injection resulted in infection of myocytes. In mice, AM were preferentially infected over DC which was opposite in the case of non-human primates. Of interest, preferential targeting of DC over AM would be an advantage for a vector-based vaccine, as AM are notoriously inefficient APC in comparison to DC^43–46^. As a proxy for the sequence of events after vaccination of humans with MVA, the non-human primate data show that directly after administration, MVA infects local APC, which in the case of aerosol delivery were CD11c^+^ MHC class II^+^ DC in the lung. These rMVA-GFP^+^ DC subsequently migrate to the draining LN, where they can present antigens via both MHC class I and II to responder cells, leading to an efficient shaping of the immune response (reviewed in ref. 47)^48^. Previously, it was shown that infection of DC with MVA induced their maturation, potentially forming the basis of direct and cross-presentation of MVA-encoded antigens by HLA class I and II^18^. Whether rMVA-GFP-infected APC present antigens to responder cells directly, or via uptake of antigen by other DC for MHC class II- and cross-presentation, was not examined in this study. Data obtained in this study show that direct targeting of professional APC could explain the excellent immunogenicity of MVA-based vaccines. Knowledge on targeting of specific cell types by MVA could guide future vaccine design and delivery strategies.

## Materials and Methods

### Ethics statement

The use of human PMBC for scientific research was approved by the Medical Ethics Committee (METC) of the Erasmus MC (permit number MEC-2015-095). All participants gave informed consent and permission for use of materials to study infectious diseases, no identifying information was published. All work described has been carried out in accordance with the code of ethics of the world medical association (declaration of Helsinki). Animal experiments were conducted in strict compliance with European guidelines (EU directive on animal testing 2010/63/EU) and the animal protocol was approved by an independent animal experimentation ethical review committee (Erasmus MC permit number EUR3293). Animal welfare was observed on a daily basis and to minimize animal suffering all animal handling was performed under light anaesthesia using 4% isoflurane in oxygen (mice) or a mixture of 10 mg/kg ketamine and 0,05 mg/kg medetomidine (ferrets and non-human primates). To antagonize the effect of medetomidine, atipamezole was administered after handling.

### Generation of rMVA-GFP

rMVA-GFP was generated by homologous recombination as described previously^49^. Briefly, MVA clonal isolate F6^1^ served as the parental virus for generating rMVA-GFP. Vector plasmid pG06-P11-GFP was used to direct insertion of GFP coding sequences under the transcriptional control of the natural VACV late promotor P11 into the deletion III site in the MVA genome. Virus stocks were generated in CEF, purified by ultracentrifugation through 36% sucrose and reconstituted in a 120 mM NaCl 10 mM Tris-HCl buffer (pH 7.4). Titre of the rMVA-GFP stock was determined by plaque assay on CEF and the construct was validated by PCR, nucleotide sequencing and transgene expression in various cell types.

### *In vitro* infections of human PBMC

PBMC were isolated from blood obtained from healthy humans and seeded into 96-wells low-adherent v-bottom plates at 50.000 cells/well. Subsequently, cells were inoculated with rMVA-GFP at incrementing MOI (range MOI 0.01–100, 3-fold titration), for 1 h, washed and incubated for 24 h in Roswell Park Memorial Institute (RPMI) 1640 medium (Lonza) supplemented with 10% heat-inactivated foetal bovine serum (HI-FBS, Greiner Bio-One), 100 μg/ml penicillin, 100U/ml streptomycin (Lonza) and 2mM L-Glutamine (Lonza) (P/S/G) at 37 °C. After 24 h, cells were harvested and stained with CD3^APC/Cy7^ (BD Pharmingen), CD11c^APC^ (BD Pharmingen), CD14^PerCP/Cy5.5^ (BD Pharmingen), CD56^PE^ (BD Pharmingen), CD20^PE/Cy7^ (Becton Dickinson) and HLA-DR^PB^ (Biolegend), in combination with LIVE/DEAD aqua fixable stain (Invitrogen). Cells were analysed on a flow cytometer (FACS Canto II) and the percentage of infected cells (GFP^+^) within the respective subsets was determined using FACS Diva software (BD Biosciences).

### *Ex vivo* infection of mouse lung explants

Lungs from mice that received IM injection with rMVA-GFP (no GFP^+^ events were detected in the lungs) were inflated with 4% low-melting 2-hydroxyethylagarose in PBS mixed with an equal part of Dulbeccos’ Modified Eagle’s Medium (DMEM, Lonza Bio-Whittaker)/Ham’s F12 medium (Gibco) supplemented with 10% HI-FBS, P/S/G and amphotericin B (1 μg/ml, institutional pharmacy). Lung slices of approximately 1mm thick were cut and used for *ex vivo* infections with rMVA-GFP as described previously^50^. In short, 24 h post-inoculation single cell suspensions were obtained by digestion (described below), and subsequently stained with CD3^PerCP^ (BD Pharmingen), CD19^PE-Cy7^ (BD Pharmingen), CD11c^APC^ (BD Pharmingen) and CD11b^APC-Cy7^ (BD Pharmingen), in combination with LIVE/DEAD aqua fixable stain. Samples were acquired on a flow cytometer (FACS Canto II) and analysed using FACS Diva software (BD Biosciences). One mock-infected lung slice was excluded from the FACS analysis due to high aspecific background.

### *In vivo* inoculation of mice with rMVA-GFP

Specified pathogen free, 6–8-week-old, female C57BL/6 mice (n = 72) were purchased from Charles River and housed in individual ventilated cage (IVC) units with access to food and water *ad libitum*. Mice were inoculated with 10^7^, 10^8^ or 10^9^ PFU rMVA-GFP in PBS administered either by IM injection in both hind legs (50 μl/leg) or IN instillation (50 μl). Mice were euthanized by cervical dislocation under isoflurane anaesthesia at 6, 24 and 48 HPA. After euthanasia, blood, ING-LN, spleen, head, lungs and trachea were obtained from all animals for analysis. In addition, the AX-LN and leg muscles were collected from IM injected animals and the MED-LN was collected from IN instilled mice.

### *In vivo* inoculation of ferrets with rMVA-GFP

Four 6–12 months old female ferrets (*Mustela Putorius furo)* were housed in negatively pressurized, HEPA-filtered BSL-3 isolator cages and provided with commercial food pellets and water *ad libitum*. Ferrets were inoculated with 10^9^ PFU rMVA-GFP in PBS via either IM injection, in both hind legs (200 µl/leg), or IT inoculation (1 ml). Ferrets were euthanized by exsanguination at 6 HPA under deep ketamine sedation (20 mg/kg body weight) (Fig. 2). Blood, ING-LN, spleen, nose, trachea, primary bronchus, lungs, BAL and TB-LN were obtained from all animals. Leg muscles were exclusively obtained from the IM injected ferrets.

### Droplet size characterization of rMVA-GFP aerosol

To characterize the aerosol droplet diameter following nebulization of rMVA-GFP, 10^9^ PFU rMVA-GFP diluted in 0.5 ml PBS was nebulized using a vibrating mesh nebulizer (Aeroneb Lab nebulizer, Aerogen Limited) as previously described^51^. The MMAD of the resulting aerosol was determined using a Cascade Impactor at 15 litres per minute vacuum flow rate (NGI, Copley Scientific).

### *In vivo* aerosol inoculation of non-human primates with rMVA-GFP

Four male, healthy cynomolgus macaques (6–8-years-old, *Macaca fascicularis*) were obtained and reused one year after a non-lethal measles vaccination and challenge experiment. All animals were housed in negatively pressurized, HEPA-filtered BSL-3 isolator cages and were inoculated with 10^9^ PFU rMVA-GFP in PBS via either IM injection in both hind legs (200 µl/leg), or by aerosol inhalation. Briefly, rMVA-GFP was administered using a nebulizer (Aeroneb Lab nebulizer, Aerogen Limited) connected to a 22 mm T-piece and using a silicone pediatric resuscitation mask (ComfortSeal silicone mask assembly, small, Monaghan Medical Corp.) as the interface with the non-human primates^26^. Using this method, 10^9^ PFU rMVA-GFP (0.5 ml) was nebulized and administered to the non-human primates. Animals were euthanized by exsanguination at 6 HPA under deep ketamine sedation (20 mg/kg body weight) (Fig. 2). Blood, inguinal LN, spleen, nasal septum, nasal concha, trachea, primary bronchus, lungs, BAL and TB-LN were collected from all animals. Leg muscles were exclusively obtained from IM injected non-human primates.

### Processing of blood, BAL and lymphoid samples

Blood samples from mice, ferrets and non-human primates were collected in Vacuette tubes containing K_3_EDTA as an anticoagulant. Pure WBC were obtained by treatment of EDTA blood with red blood cell (RBC) lysis buffer (Roche diagnostics). A BAL was performed post-mortem in ferrets and non-human primates by direct infusion of 10 ml PBS into the lungs, followed by immediate recovery of the fluid. Lymphoid organs were collected from animals during necropsy in Iscove’s Modified Dulbecco’s Medium (IMDM, Lonza Bio-Whittaker) supplemented with 5% HI-FBS and P/S/G. Single cell suspensions were generated by using 100 μm cell strainers (Falcon). Spleen single cell suspensions were subsequently treated with RBC lysis buffer in order to remove erythrocytes. Collectively, WBC, BAL cells and single cell suspensions from lymphoid organs were directly analysed for presence of GFP by flow cytometry on a FACS Canto II (BD Biosciences). GFP^+^ samples were subsequently stained in order to phenotype GFP^+^ events (see below). Mouse ING-LN and WBC samples after IM injection (n = 1), spleen after IN instillation (n = 1) were excluded from FACS analysis due to high background signal.

### Preparation and processing of lungs

Mouse lungs were either fixed by inflation with 10% formalin or complete single cell suspensions were prepared by digestion. Formalin-fixed tissues were subsequently analysed by immunohistochemistry and/or dual immunofluorescent staining. Single cell suspensions were prepared by treatment with 300 U/ml collagenase type I (Invitrogen) and 0.15 mg/ml DNase (Roche diagnostics) diluted in Hank’s Balanced Salt Solution (HBSS, Gibco) for 1 h at 37 °C, followed by filtration over 100 μm cell strainers. Excess erythrocytes were removed by treatment with RBC lysis buffer if necessary. Single cell suspensions were used directly for detection of GFP by flow cytometry. GFP^+^ samples were subsequently stained in order to phenotype GFP^+^ events (see below).

Ferret and non-human primate lungs were inflated with 4% low-melting 2-hydroxyethylagarose in PBS mixed with an equal part of DMEM/Ham’s F12 medium (Gibco) supplemented with 10% HI-FBS, P/S/G and amphotericin B. Lung slices of approximately 1mm thick were cut and used for direct analysis of GFP fluorescence using CLSM. After direct detection of GFP, lung slices were fixed in 10% formalin, paraffin embedded and subsequently analysed by immunohistochemistry and/or dual immunofluorescent staining.

### Phenotyping by flow cytometry

Freshly isolated WBC, BAL cells and single cell suspensions prepared from lymphoid organs or the lungs were analysed directly for GFP expression using a FACS Canto II flow cytometer and FACS Diva software (BD Bioscience). Approximately 1 × 10^6^ events were obtained per sample to allow detection of low frequent GFP^+^ populations. Samples positive for GFP were subsequently stained to phenotype the GFP^+^ cells. Cells obtained from mouse lungs were subdivided into DC, eosinophils, AM, neutrophils, IMφ, CD4^+^ and CD8^+^ T-lymphocytes, B-lymphocytes and NK cells by blocking Fc receptors with 2.4G2 and staining with Siglec-F^PE^ (BD), CD3^PE/CF594^ (BD), CD19^PerCP/Cy5.5^ (eBioscience), CD8^PE/Cy7^ (eBioscience), CD11c^EF450^ (eBioscience), CD4^BV605^ (BD), MHCII^BV650^ (BD), CD45^BV711^ (BD), F4/80^Bi^°^tin^ (eBioscience) followed by Streptavidine^BV786^ (BD), NK1.1^APC^ (BD), CD11b^AF700^ (eBioscience) and Gr-1^APC/EF780^ (eBioscience). BAL cells obtained from non-human primates were subdivided into DC, eosinophils, AM, neutrophils, monocytes, epithelial cells, CD4^+^ and CD8^+^ T-lymphocytes and B-lymphocytes by blocking Fc receptors with human serum and staining with CD33^PE^ (eBioscience), CD19^PE/TXR^ (Beckman Coulter), CD16^PerCP/Cy5.5^ (BD), CD11b^PE/Cy7^ (BD), CD8a^EF450^ (eBioscience), CD68^Biotin^ (Biolegend) followed by Streptavidine^BV650^ (BD), MHCII^BV711^ (BD), CD45^BV786^ (BD), Siglec-8^APC^ (Biolegend), CD11c^AF700^ (BD) and CD3^APC/Cy7^ (BD). For both mice and non-human primates, dead cells were disregarded in the analysis by excluding cells stained with LIVE/DEAD aqua fixable stain and the percentage GFP^+^ cells within each cell population was determined using a FACS LSR II (BD Biosciences).

### Tissue preparation for immunohistochemistry

To determine the tropism of rMVA-GFP in the upper respiratory tract, complete mouse heads were fixed in 10% formalin. Heads were subsequently decalcified for eight days using a 10% EDTA buffer, split in two halves, and decalcification was continued for another two days, followed by embedding in paraffin. To determine cellular tropism in the upper respiratory tract from ferrets and non-human primates, nasal tissue from ferrets was harvested and formalin fixed, and the nasal septa and concha from non-human primates were harvested and fixed. Tissues were decalcified before embedding into paraffin if required. Furthermore, in addition to analysis of the nasal cavities and lungs, the trachea and primary bronchi from all animals were collected in 10% formalin. To determine the tropism of rMVA-GFP in the muscles, hind and front leg muscles were harvested in PBS. If possible, the exact site of the rMVA-GFP injection was harvested. Subsequently, slices were cut for direct examination of GFP fluorescence using CLSM. Positive slices were formalin-fixed and paraffin-embedded, followed by immunohistochemistry and/or dual immunofluorescence staining. In all animal models, the front leg muscles formed the appropriate negative control for the inoculated hind leg muscle tissues.

### Direct confocal laser scanning microscopy (CLSM)

Slices obtained from muscle tissues or agarose-inflated lungs were directly analysed for GFP fluorescence using CLSM with a LSM700 system fitted on an Axio Observer Z1 inverted microscope (Zeiss). Images and videos were generated using Zen software. GFP^+^ muscle from mice and lungs slices from cynomolgus macaques were transferred to 4% (w/v) paraformaldehyde in PBS, permeabilized with 0.1% (v/v) Triton-X100 for 30 min and subsequently counterstained for nuclei with TO-PRO-3 (Invitrogen).

### Immunohistochemistry and dual immunofluorescence analysis of formalin-fixed tissues

Sections from formalin-fixed paraffin-embedded tissues were cut (3 µm) and deparaffinised. In order to detect GFP, antigen retrieval was performed in 10 mM citrate buffer. For histological evaluation, samples were subsequently incubated overnight with anti-GFP polyclonal rabbit antibody (Invitrogen) at 4 °C, followed by incubation with a secondary goat-anti-rabbit-biotin antibody (DAKO) and streptavidin-HRP (DAKO). GFP was detected using 3-amino-9-ethylcarbazole (AEC) as substrate. The slides were counterstained with haematoxylin^51^.

On mouse and non-human primate hind leg muscle and lung tissues dual immunofluorescence stainings were performed. After antigen retrieval in citrate buffer, tissues were incubated with 10% normal goat serum (Bio-Connect) in PBS. Mouse tissues were stained with rabbit anti-GFP (Invitrogen) or rabbit IgG isotype (R&D Systems) followed by goat-anti-rabbit Alexa Fluor (AF)488 (Life Technologies). Non-human primate tissues were stained with rabbit anti-GFP in combination with mouse-derived antibodies: anti-desmin (Abcam), anti-cytokeratin (DAKO), anti-Mac387 (AbD Serotec), anti-MHC class II (HLA-DP/DQ/DR, DAKO), anti-CD11c (Novocastra Biosystems Newcastle Ltd) or anti-CD20 (DAKO). As a negative control, lung slices were stained with rabbit IgG isotype in combination with either mouse IgG1 or IgG2a isotype (R&D Systems). Subsequently, tissues were stained with goat-anti-rabbit Alexa Fluor (AF)488 and goat-anti-mouse AF549 (Invitrogen). All samples were treated with ProLong Gold antifade reagent with DAPI (Life Technologies) before analysis using CLSM with a LSM700 system fitted on an Axio Observer Z1 inverted microscope (Zeiss). Images were analysed using Zen software.

### Data availability

All data generated or analysed during this study are included in this published article (and its Supplementary Information files).

## Electronic supplementary material

Supplementary information

Supplemental Movie 1

Supplemental Movie 5
