# Supplementary material for: Modified Vaccinia Virus Ankara Preferentially Targets Antigen Presenting Cells In Vitro, Ex Vivo and In Vivo
Source: Sci Rep. 2017 Aug 17;7:8580. doi: 10.1038/s41598-017-08719-y (PMC5561217; doi:10.1038/s41598-017-08719-y)
Supplement: Supplementary file 1 — Supplementary information [file 41598_2017_8719_MOESM1_ESM.pdf]

Modified Vaccinia Virus Ankara Preferentially Targets Antigen Presenting Cells *In Vitro*, *Ex Vivo* and *In Vivo*

Arwen F Altenburg[1], Carolien E van de Sandt[1*], Bobby WS Li[2], Ronan J MacLoughlin[3], Ron AM Fouchier[1], Geert van Amerongen[4], Asisa Volz[5,6], Rudi W Hendriks[2], Rik L de Swart[1], Gerd Sutter[5,6], Guus F Rimmelzwaan[1], Rory D de Vries[1#]*

[1] Department of Viroscience, Postgraduate School of Molecular Medicine, Erasmus MC, Rotterdam, the Netherlands

[2] Department of Pulmonary Medicine, Erasmus MC, Rotterdam, the Netherlands

[3] Aerogen Ltd, IDA Business Park, Dangan, Galway, Ireland

[4] ViroClinics Biosciences BV, Rotterdam, the Netherlands

[5] Institute for Infectious Diseases and Zoonoses, LMU University of Munich, Munich, Germany

[6] German Centre for Infection Research (DZIF)

[*] Authors contributed equally

[#] **Corresponding author:** Dr. Rory D de Vries, Department of Viroscience, Erasmus MC, PO Box 2040, 3000 CA Rotterdam, the Netherlands, email address: r.d.devries@erasmusmc.nl

[MOVIE]

Supplementary Figure 1. CLSM 3D render of mouse hind leg muscle. Z-stack of a hind leg muscle slice from a IM rMVA-GFP injected mouse was obtained by CLSM. A 3D render of the maximum intensity projection was generated using the Zen software. GPF = green. Nucleus = red.

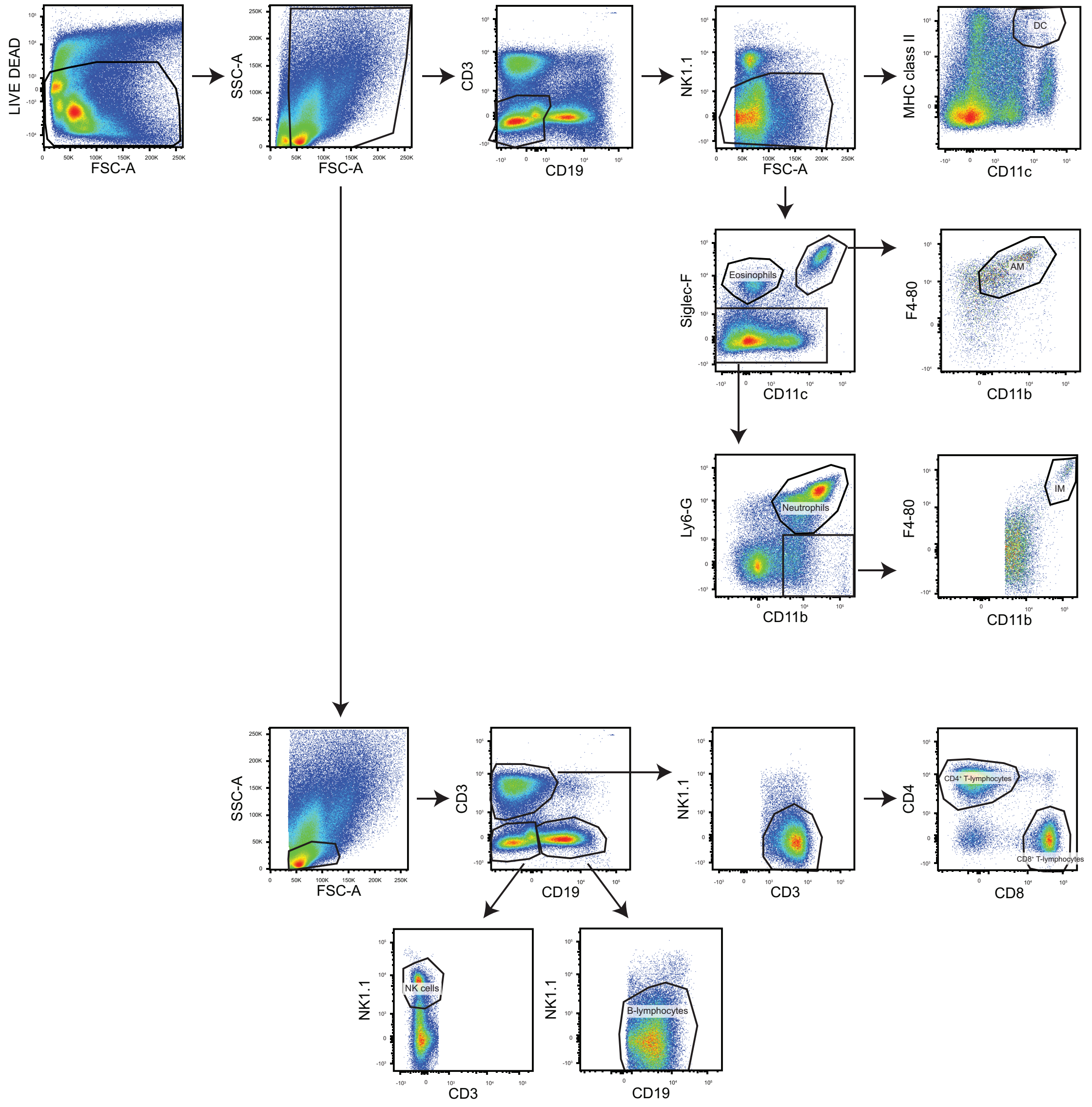

Supplementary Figure 2. Gating strategy to define populations for phenotypic analysis of GFP$^+$ cells in the lungs of mice. Live cells were gated followed by selection of non-lymphocytes and lymphocytes based on the forward / sideward scatter. Subsequently, CD3$^-$ CD19$^-$ NK1.1$^-$ cells were selected in the non-lymphocyte population. MHC class II$^+$ CD11c$^+$ cells were defined as DC and Siglec-8$^+$ CD11c$^-$ were classified as eosinophils. Siglec-8$^+$ CD11c$^+$ F4-80$^+$ CD11b$^+$ cells were identified as alveolar macrophages (AM). Siglec-8-negative cells were further subdivided into a Ly6-G$^+$ CD11b$^+$ neutrophil population and Ly6-G$^-$ CD11b$^+$ F4-80$^+$ interstitial macrophages (IM).

Following selection of lymphocytes in the forward / sideward scatterplot, CD3$^+$ CD19$^-$ NK1.1$^-$ cells were selected and divided into CD4$^+$ and CD8$^+$ T-lymphocytes. Furthermore, CD3$^-$ CD19$^-$ NK1.1$^+$ cells were defined as NK cells and CD3$^-$ CD19$^+$ NK1.1$^-$ cells were defined as B-lymphocytes.

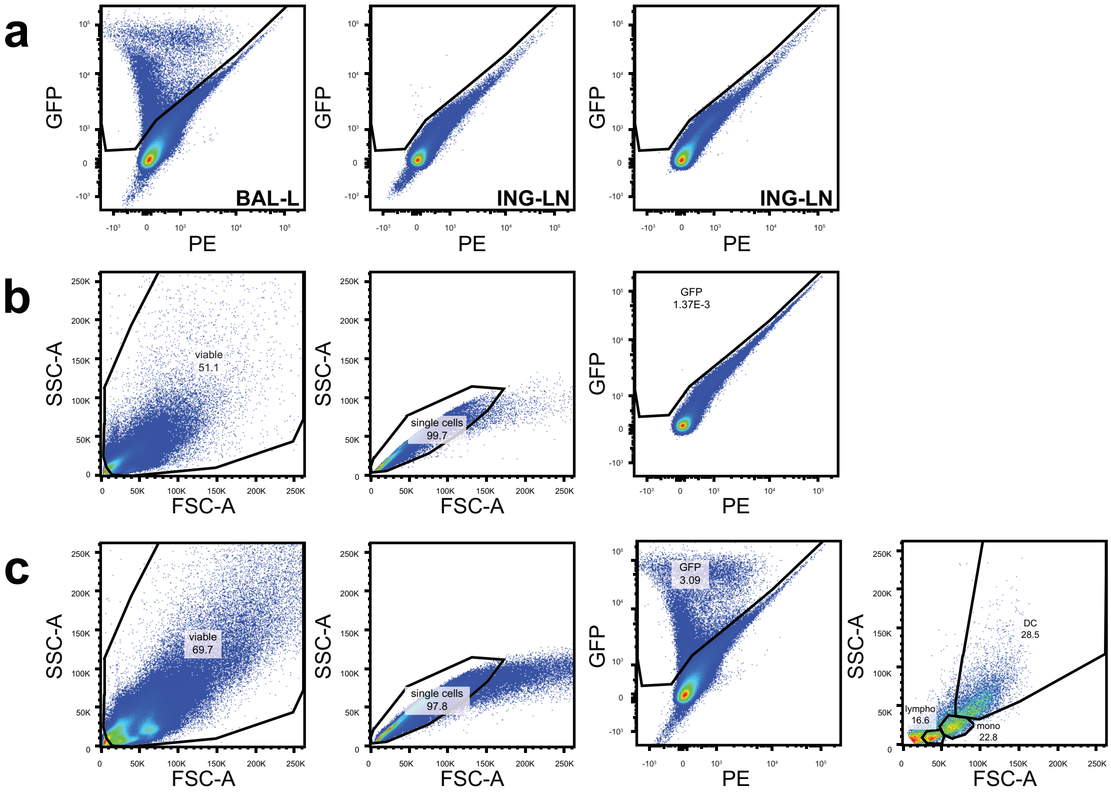

Supplementary Figure 3. Gating strategy to detect GFP+ cells in ferret tissues. (**A**) Example of detection of GFP+ cells in unstained single cell suspensions from different ferret tissues. Detection of GFP+ cells in a sample containing abundant GFP+ cells (panel 1, BAL left side after IT inoculation), background level GFP+ cells (panel 2, ING-LN after IM injection) or no GFP+ cells (panel 3, ING-LN after IT inoculation) is shown. (**B-C**) Gating strategy to define GFP+ DC-like, monocyte-like and lymphocyte-like cell populations in ferret tissues. As an example, the gating strategy of BAL is shown after IM injection (**B**, negative control) or IT inoculation (**C**) with rMVA-GFP. First, viable cells were gated followed by selection of single cells. Next, all GFP+ cells were selected after which lymphocyte-like, monocyte-like or DC-like cell populations were reversely gated in the scatter plot. Gate name and percentage of events are indicated in each gate.

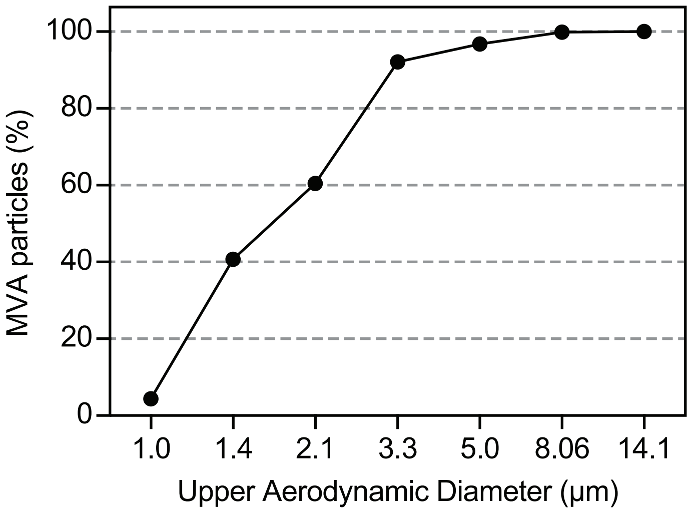

Supplementary Figure 4. Droplet size characterization of rMVA-GFP aerosol. rMVA-GFP was nebulized and several droplet fractions of increasing size between 0.98 and 14.1μm in diameter were collected using a cascade impactor. Cumulative distribution of rMVA-GFP particles across the range of droplet diameters is shown.

[MOVIE]

Supplementary Figure 5. CLSM 3D render of macaque lung. Z-stack was obtained of a lung slice from a macaque that received rMVA-GFP via AER inhalation. A 3D render of the maximum intensity projection was generated using Zen software. GPF = green. Nucleus = red.

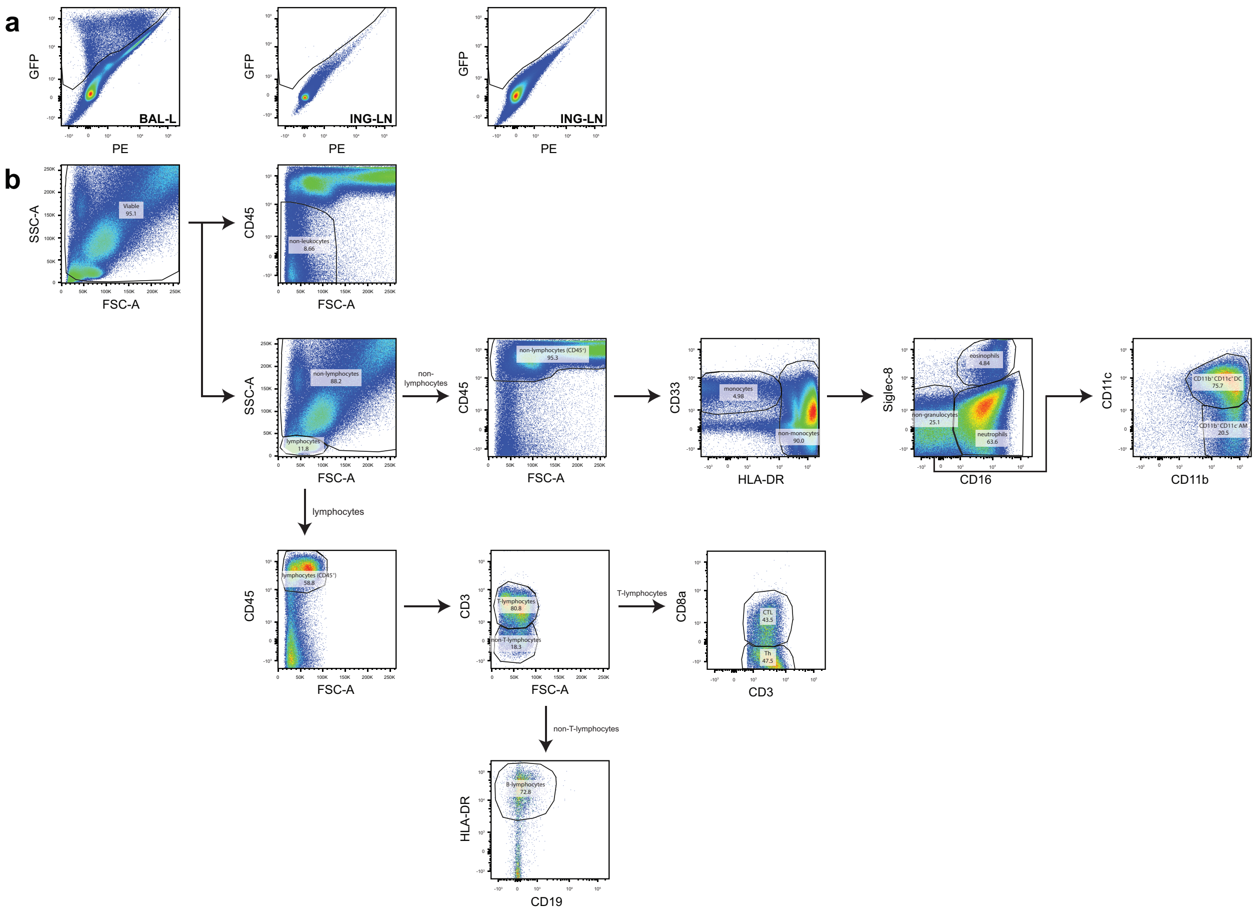

Supplementary Figure 6. Gating strategy to detect GFP$^+$ cells in macaque tissues. (**A**) Example of detection of GFP$^+$ cells in unstained single cell suspensions from different non-human primate tissues. Detection of GFP$^+$ cells in a sample containing abundant GFP$^+$ cells (panel 1, BAL left side after AER inhalation), background level GFP$^+$ cells (panel 2, ING-LN after IM injection) or no GFP$^+$ cells (panel 3, ING-LN after IT inoculation) is shown. (**B**) Gating strategy to define populations for phenotypic analysis of GFP$^+$ cells in BAL of macaques. Viable cells were selected after which non-leukocyte (*e.g.* CD45$^-$ epithelial cells) were gated. Viable cells were further discriminated into lymphocytes and non-lymphocytes based on FSC / SSC plot. CD45$^+$ cells outside the lymphogate were selected in which the CD33$^+$ monocytes were gated. The HLA-

DR$^+$ population was divided into Siglec-8$^+$ CD16$^+$ eosinophils, Siglec-8$^-$ CD16$^+$ neutrophils and Siglec-8$^-$ CD16$^-$ non-granulocytes, which were further phenotyped into CD11b$^+$ CD11c$^+$ DC or CD11b$^+$ CD11c$^-$ AM. The CD45$^+$ lymphocytes were divided into CD3$^+$ T-lymphocytes, further discriminated into CD8$^+$ cytotoxic T-lymphocytes (CTL) and CD8$^-$ T helper (Th)-lymphocytes, and CD3$^-$ HLA-DR$^+$ B-lymphocytes. In this example CD19 staining did not work, thus was not included in the analysis. Gate name and percentage of events is indicated in each gate.
